# Dermoscopic Correlation of an Eccentric Case of Kindler Syndrome

**DOI:** 10.7759/cureus.58433

**Published:** 2024-04-16

**Authors:** Sarthak Bansal, Sanjeev Gupta, Shachi Jain, Aayush Gupta

**Affiliations:** 1 Dermatology, Dr. D. Y. Patil Medical College, Hospital & Research Centre, Dr. D. Y. Patil Vidyapeeth, Pune, IND

**Keywords:** hyperpigmentation, photosensitivity, poikiloderma, dermoscopy image analysis, kindler syndrome

## Abstract

Kindler syndrome (KS) is a rare autosomal recessive skin condition. The FERMT1 gene mutates and causes symptoms such as blistering and epidermal atrophy, as well as an increased risk of cancer and poor wound healing. A male in his 20s sought treatment for his hyper-hypopigmentation over the body with poikiloderma of the face with thin wrinkled cigarette paper skin in association with photosensitivity. He gave a history of developing blisters all over the body during his childhood, which formed raw areas and eventually healed forming atrophic scars. The objective is to assess the correlation of clinical findings with dermoscopy in a case of KS. KS is a rare disorder with poikiloderma, photosensitivity, and acral bullae in infancy as predominant features. Dermoscopy proves to be a useful tool in the diagnosis of this rare disorder as it helps in the identification of poikiloderma, adermatoglyphia, and cigarette paper scarring.

## Introduction

Kindler syndrome (KS) is a rare autosomal recessive dermatological condition that is characterized by acral blistering in infancy and childhood over trauma-prone areas, photosensitivity, progressive poikiloderma, and cutaneous atrophy [1]. The molecular pathogenesis of KS is linked to loss-of-function mutations in an actin cytoskeleton-associated protein that is currently known as fermitin family homolog 1, which is encoded by the gene FERMT1. The structural pathology of this protein is different from other forms of epidermolysis bullosa in which the keratin intermediate filament-hemidesmosome network and the extracellular matrix are disrupted. Through focal adhesions, this protein mediates anchoring between the actin cytoskeleton and the extracellular matrix [2]. For the diagnosis of KS, a diagnostic criterion is used, which comprises major criteria, minor criteria, and associated findings. The major criteria comprise acral blistering in infancy and childhood, skin atrophy, gingival fragility and/or swelling, abnormal photosensitivity, and progressive poikiloderma whereas the minor criteria include esophageal, anal, urethral, or laryngeal stenosis. Other related findings include skeletal anomalies, poor dentition/dental caries/periodontitis, nail dystrophy, ectropion of the lower lid, palmoplantar keratoderma, pseudoainhum, leukokeratosis of the lips, squamous cell carcinoma, and anhidrosis/hypohidrosis. When all four of the major criteria are met, the diagnosis of KS is definite. The diagnosis is probable if three major criteria and two minor criteria are met, and likely if two major criteria, two minor criteria, and related symptoms are present [3].

## Case presentation

**Figure 1:**
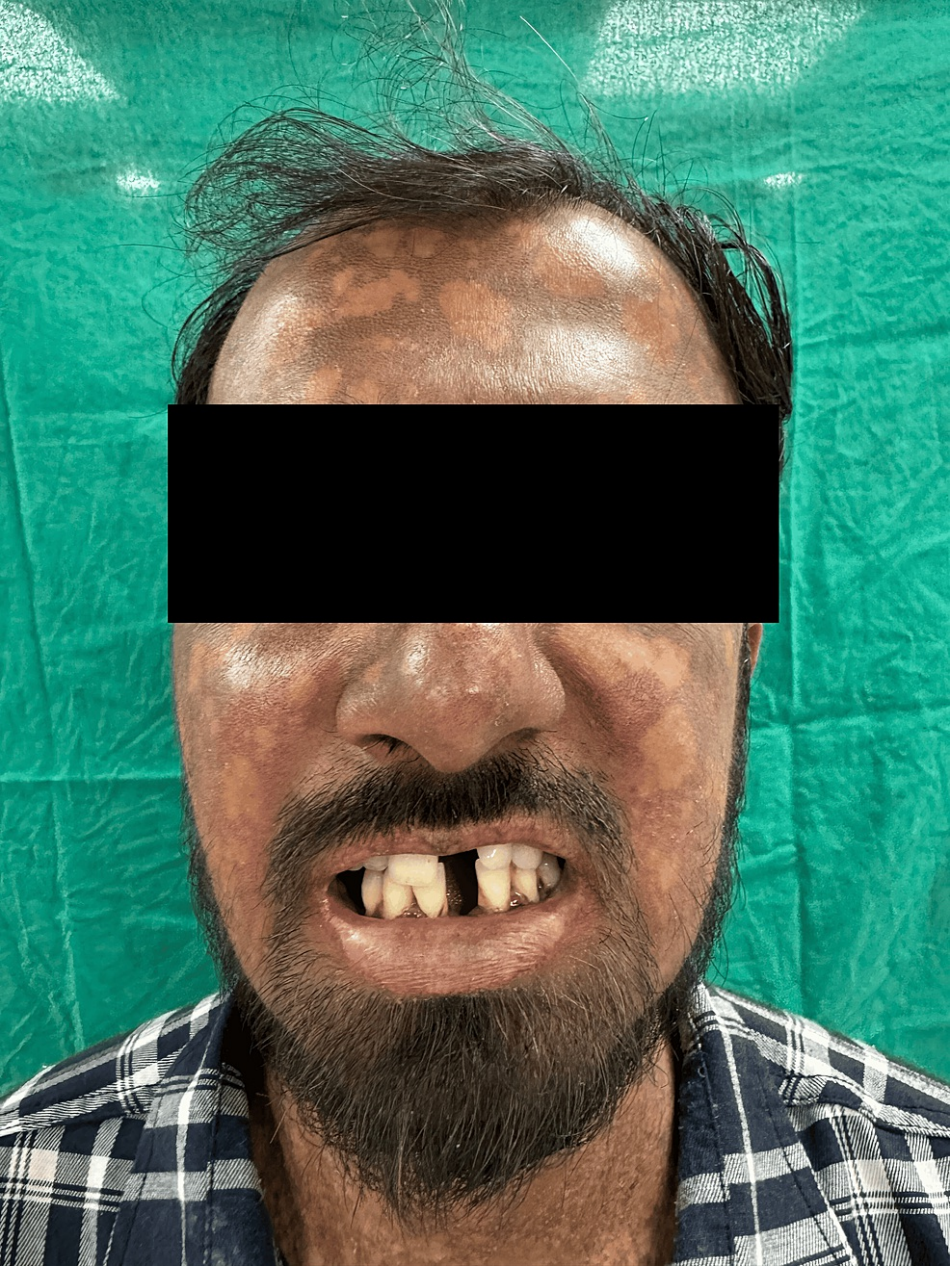
Poikiloderma of the face Telangiectasia, dyspigmentation, and atrophy with thin wrinkled cigarette paper appearance.

**Figure 2:**
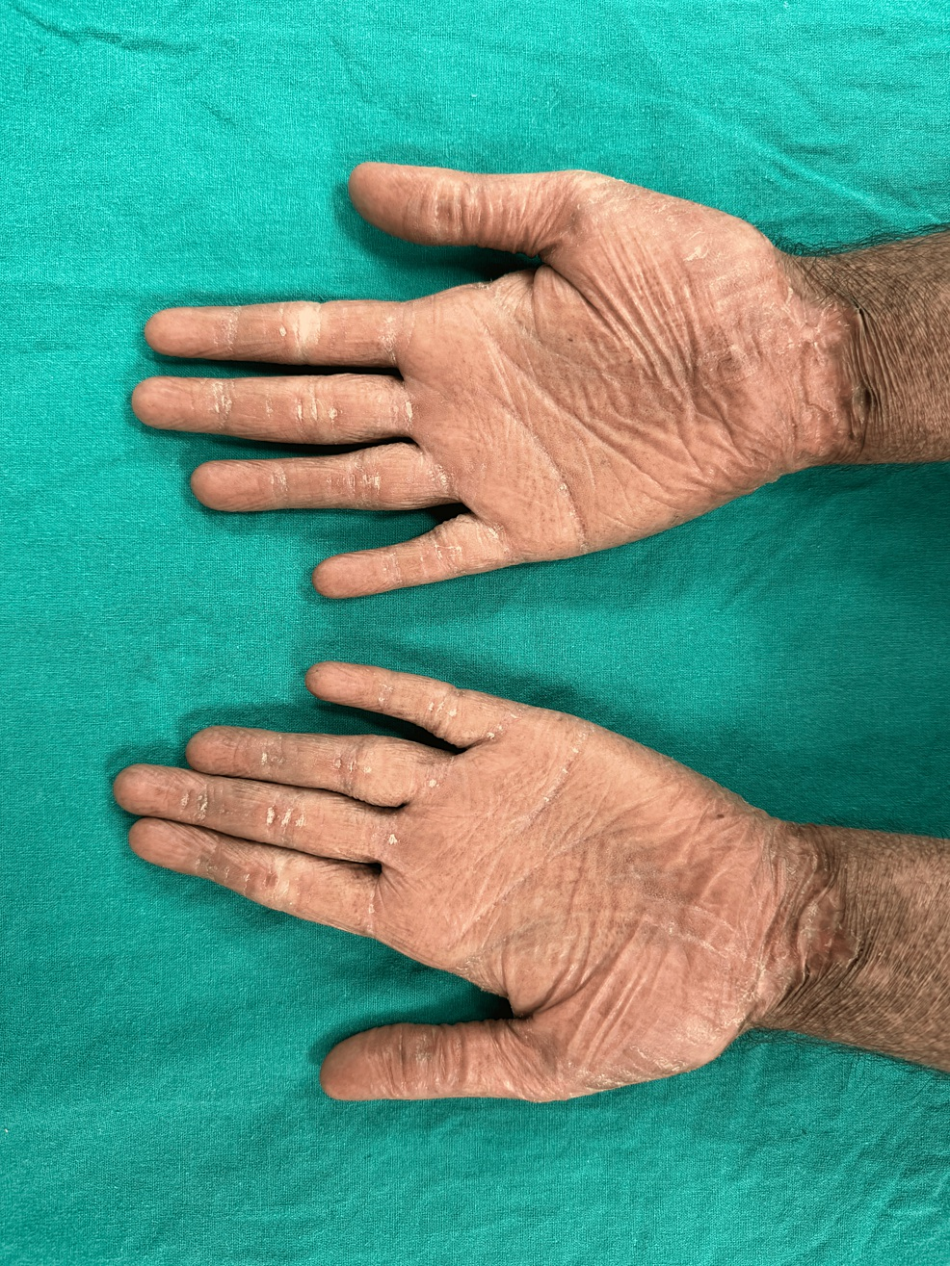
Hands The palms of both hands are waxy in appearance with atrophy and loss of dermatoglyphics.

**Figure 3:**
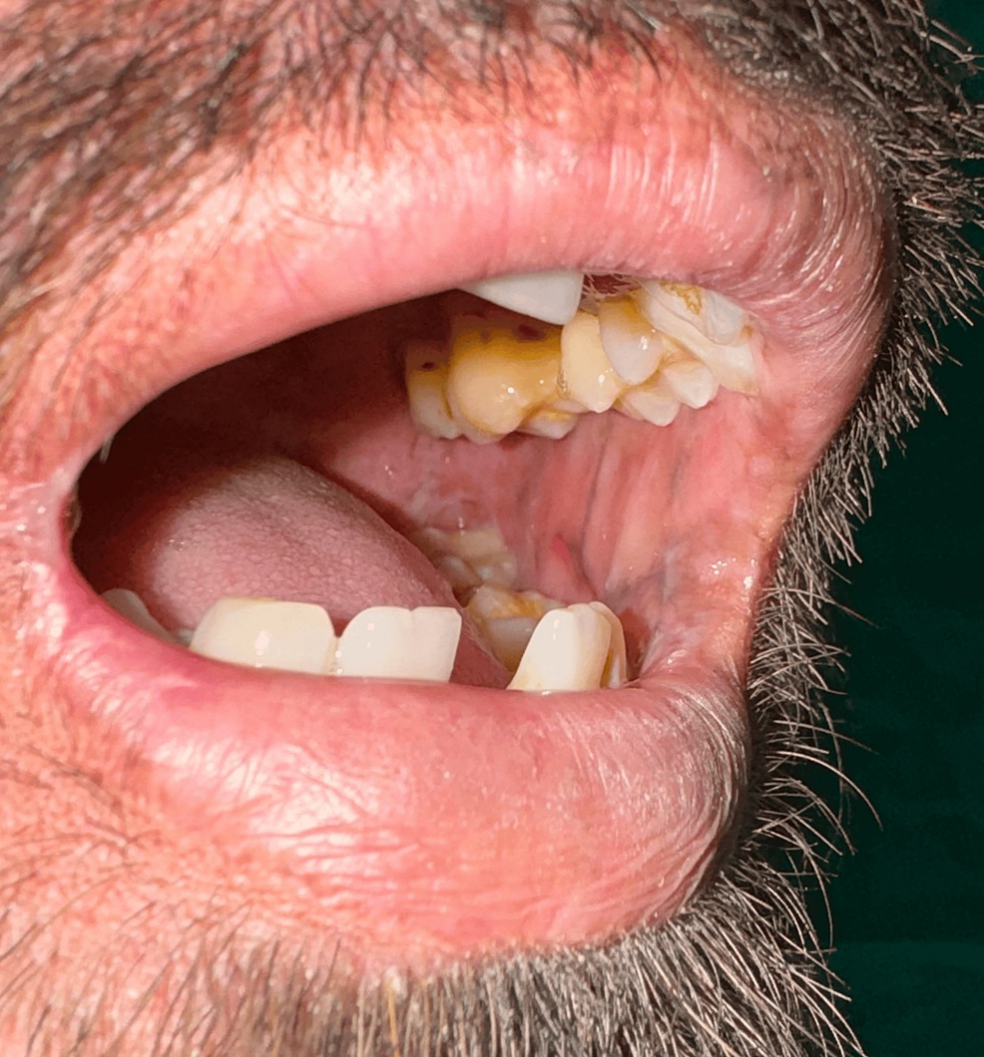
Oral cavity The oral cavity shows leukokeratosis with premature loss of teeth.

A male in his mid-20s took a dermatology consult from a tertiary care hospital for complaints of dusky-colored lesions all over the body that were associated with photosensitivity. The patient gave a history of multiple clear fluid-filled blisters all over the body, especially over extensors and trauma-prone areas, throughout his childhood, which then improved with age. They eventually healed forming atrophic scars with hyper and hypopigmentation. He developed poikiloderma of the face, which is characterized by telangiectasia, dyspigmentation, and atrophy with thin wrinkled cigarette paper appearance of skin (Figure 1). His palms and soles were waxy in appearance with loss of dermatoglyphics and atrophy (Figure 2). Nail examination demonstrated onycholysis, subungual hyperkeratosis, and nail dystrophy. His oral cavity revealed leukokeratosis with premature loss of teeth (Figure 3). There was no abnormality detected on the genital examination. On systemic examination, there was no abnormality detected. His blood and urine analyses were within normal limits. Genetic testing was not performed since the patient could not afford it. Based on the history and clinical features, the patient was diagnosed with KS. As there is no such treatment for this condition, the goal of management is to alleviate the symptoms, hence the patient was given emollients and photoprotection.

**Figure 4:**
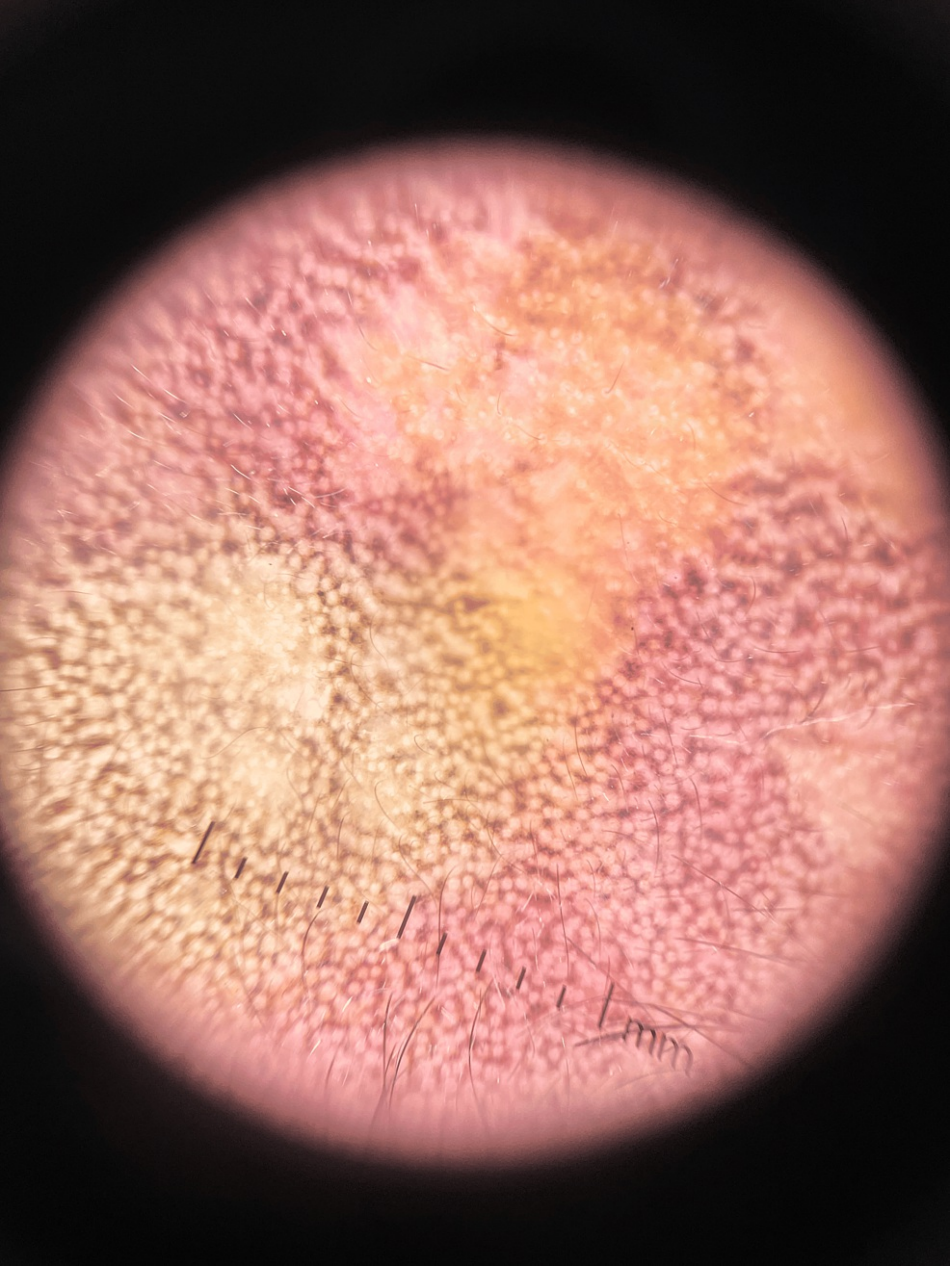
Dermoscopy of the face Dermoscopy depicts blue and grey pigment with interspersed normal pigment over the affected area in a pseudoreticular pattern with telangiectasia within the background erythema.

Dermoscopy depicted blue and grey pigment with interspersed normal pigment over the affected area in a pseudoreticular pattern with telangiectasia within the background erythema. The presence of islands of normal skin within the affected area could be probably due to revertant mosaicism (Figure 4) [4,5].

His skin biopsy revealed hyperkeratotic as well as atrophic epidermis with focal basal cell vacuolation resulting in subepidermal separation. The dermis revealed dilated capillaries, mild perivascular lymphocytic infiltrate, and marked pigment incontinence with thickened collagen bundles and atrophic adnexal structures.

## Discussion

KS is a rare subtype of inherited epidermolysis bullosa associated with FERMT 1 (KIND1) gene mutation. It is thought to occur via a loss-of-function mechanism [6]. It is unclear how the FERMT1 gene mutates and causes symptoms such as blistering and eventually epidermal atrophy. It is also hypothesized to be associated with an increased risk of squamous cell carcinoma of the skin and poor wound healing [6]. There is currently no proven treatment for KS. The goals of management are to alleviate symptoms while minimizing complications. The presence of a healthy patch of skin among damaged skin, confirmed by immunostaining of normal skin, indicates revertant mosaicism, which is a genetic condition that causes spontaneous partial or complete correction of the impaired phenotype [5]. This phenomenon is often observed in several inherited conditions and represents a potential future treatment modality. Patients with KS should be seen by a multidisciplinary team in a facility that has expertise in treating children with skin fragility, including dermatologists, ophthalmologists, dentists, and dietitians. The patients are managed with the use of emollients and sunscreen. Mucosal involvement necessitates maintaining adequate oral hygiene to prevent periodontal disease and lubricating the cornea to prevent ocular injury [7]. Detection of loss of function mutation in the KIND1 gene establishes the diagnosis. Genetic analysis is usually not done due to monetary constraints [8,9].

## Conclusions

KS is usually diagnosed based on the correlation of clinical and dermoscopic findings with histopathological changes. Dermoscopy although may not be required for confirmation of diagnosis of this rare disorder, it guides us in delineating certain clinical signs, which are not easily visible to the naked eye. It helps in the identification of features such as adermatoglyphia in documenting poikiloderma with wrinkled cigarette paper scarring and signs of revertant mosaicism. Dermoscopic findings along with the history and clinical features help in determining the diagnosis, which can further be confirmed by histopathological changes.
